# Dr. Kinglake, on Reduced Temperature

**Published:** 1803-10-01

**Authors:** Robert Kinglake

**Affiliations:** Taunton


### *To the Editors of the Medical and Physical Journal.*

GENTLEMEN,

THE communication in your last number, subscribed E. O. on the salutary efficacy of reduced temperature in rheumatic or gouty affection, is so pointedly in support of the treatment proposed by me, for the prompt and effectual cure of that malady, as to induce me to trouble you to request of the author his real signature, that no possible suspicion may attach to its authenticity from its appearing anonymously.

Such a detail of facts is truly valuable, and should possess every external claim to implicit admission as correct medical evidence.

A knowledge of the intelligent author's name will enable me to incorporate his case with many others, in an appendix to a work on Gout, now preparing by me for speedy publication. In the mean time, the transmission, either to your Journal or to myself, of any farther experience on this interesting subject, cannot fail to extend and improve the object of my inquiry.

The success of the treatment under my own direction continues to exceed my most sanguine expectation, and promises soon to acquire it a very general and confident adoption.

I am, &c.

*Taunton, Sept.* 8, 1803. ROBERT KINGLAKE.

